# Exploratory Determined Correlates of Physical Activity in Children and Adolescents: The MoMo Study

**DOI:** 10.3390/ijerph16030415

**Published:** 2019-01-31

**Authors:** Steffen CE Schmidt, Jennifer Schneider, Anne Kerstin Reimers, Claudia Niessner, Alexander Woll

**Affiliations:** 1 Institute of Sports and Sports Science, Karlsruhe Institute of Technology, 76131 Karlsruhe, Germany; jennifer.schneider@student.kit.edu (J.S.); Claudia.Niessner@kit.edu (C.N.); Alexander.Woll@kit.edu (A.W.); 2 Institute of Human Movement Science and Health, Faculty of Behavioral and Social Sciences, Technical University of Chemnitz, 09111 Chemnitz, Germany; anne.reimers@hsw.tu-chemnitz.de

**Keywords:** physical activity, correlates, children, adolescents, MoMo study, LASSO

## Abstract

*Background*: Physical activity is an important contributor to reducing the risk for a variety of diseases. Understanding why people are physically active contributes to evidence-based planning of public health interventions because successful actions will target factors known to be related to physical activity (PA). Therefore the aim of this study is to identify the most meaningful correlates of PA in children and adolescents using a large, representative data set. *Methods*: Among *n* = 3539 (1801 boys) 6 to 17-year-old participants of the German representative Motorik-Modul baseline study (2003–2006) a total of 1154 different demographic, psychological, behavioral, biological, social and environmental factors were ranked according to their power of predicting PA using least absolute shrinkage and selection operator (LASSO) regressions. *Results*: A total of 18 (in girls) and 19 (in boys) important PA predictors from different, personal, social and environmental factors have been identified and ranked by LASSO. Peer modeling and physical self-concept were identified as the strongest correlates of PA in both boys and girls. *Conclusions*: The results confirm that PA interventions must target changes in different categories of PA correlates, but we suggest to focus particularly on the social environment and physical self-concept for interventions targeting children and adolescents in Germany nowadays. We also strongly recommend to repeatedly track correlates of PA, at least every 10 years, from representative samples in order to tailor contemporary PA interventions.

## 1. Introduction

PA aids healthy development throughout childhood [1] and reduces the risk of chronic diseases like type 2 diabetes or obesity [2] and mental disorders like reduced self-esteem [2,3]. Currently, the World Health Organization recommends at least 60 min of moderate to vigorous physical activity (MVPA) daily for children and adolescents [4]. Recent research shows that youths who fulfill these recommendations benefit in many health indicators [5]. However, worldwide a large proportion of young people do not meet this recommendation [6]. In Germany, only 17.4% of boys and 13.1% of girls aged 4 to 17 meet them [7]. Against the background that regular PA is particularly important in childhood and adolescence, these numbers are alarming. Youth’s PA patterns are tracking into adulthood [8,9] and PA is especially relevant for healthy development in youth which has lifelong consequences for both physical and mental health [10].

As shown in many studies, overall girls are less physically active than boys [7,11,12]. Gender differences favoring boys are especially prevalent in regard to structured PA and sports or vigorous PA, respectively [13,14,15]. While on the one hand, gender differences in PA can be explained by gender-related social constructive theories such as Social Cognitive Theory [16], on the other hand, biological theories assume that differences between boys and girls are the results of genetics, genes, and chromosomes [17]. Thus, differences in PA between boys and girls probably arise from various factors pertaining to biological and environmental constitution. This assumption has been fed into social-ecological models indicating multifaceted and interactive effects of various factors that determine health behaviors like PA [18]. In boys and girls, different factors with different levels of influence (intrapersonal, interpersonal, organizational, community, policy) may affect PA [19,20].

Until now, research on correlates and determinants of PA in youth guided the development and conduction of intervention studies that aimed to enhance PA and to prevent inactive lifestyles in youth [21,22,23]. However, many intervention programs did not effectively promote PA. Furthermore, despite the call for the promotion of PA especially in girls, PA promotion programs failed to eradicate gender differences in PA levels in children and adolescents [24]. This can potentially be attributed to the lack of gender-sensitivity of intervention programs neglecting the unique needs of boys and girls and potential similarities and differences in underlying mechanisms responsible for the success or failure of the intervention program.

Thus, it is necessary to better understand the key factors that are related to PA behavior by taking gender differences into account. However, despite assumptions of socio-ecological models and the need for comprehensive analysis of a wide range of potential correlates from different levels [25], studies just focus on a specific subsection of variables such as environmental factors [26,27]. As a consequence, the relative relevance of single correlates remained unclear and misleading conclusions might have been drawn. Additionally, gender differences in correlates have rarely been studied in large representative samples that facilitate the identification of relevant factors promoting or hindering PA in boys and girls, respectively.

The aim of the present study was to identify the most meaningful correlates of PA in German boys and girls. We approached this by ranking a wide range of possible personal, and environmental variables from a large representative sample of children and adolescents according to their power of predicting PA in least absolute shrinkage and selection operator (LASSO) regressions.

## 2. Participants and Research Methods

### 2.1. Design and Participants

The MoMo study [28] is an in-depth study of the German Health Interview and Examination Survey for Children and Adolescents (KiGGS) conducted by the Robert Koch Institute (RKI). The aim of the MoMo study is to gather nationally representative data on physical fitness, PA and health parameters of children and adolescents.

To ensure a diverse sample of children and adolescents with primary residence in Germany, the RKI and the German Centre for Surveys, Methods, and Analysis (GESIS, formerly ZUMA) drew a nationwide stratified multi-stage probability sample with three evaluation levels at KiGGS baseline [29,30]. First, a systematic sample of 167 primary sampling units was selected from an inventory of German communities stratified according to the BIK classification system that measures the level of urbanization and geographic distribution [31]. Second, an age-stratified sample of randomly selected children and adolescents, with a total of 17,641 participants aged 0–17 years, was drawn from the official registers of local residents [30]. Third, 7866 children and adolescents aged 4–17 years were randomly assigned to the MoMo baseline sample. Of these children and adolescents, 4528 (57.6%) ultimately participated in the MoMo baseline study from 2003 to 2006. From those, the 6 to 17 year-olds were selected for the present study (*n* = 3539: 1801 males).

### 2.2. Physical Activity Measurement

The MoMo Physical Activity Questionnaire (MoMo-PAQ) was used to assess self-reported habitual PA in different settings [32]. The MoMo-PAQ consists of 28 items to obtain the frequency, duration, intensity, and setting of PA retrospectively during a normal week. Data obtained with the MoMo-PAQ are sufficiently reliable (test-retest reliability: ICC = 0.68) and validated against accelerometers [32].

Participants were able to name up to four different activities they perform in sports clubs. For each activity the type of activity (e.g., soccer), duration (minutes per session), frequency (times per week) and time throughout the year (months per year) were assessed. From those items, average minutes per week with PA at sports clubs were calculated.

Additionally, up to four unorganized, leisure-time sports activities were assessed by three items each: type (e.g., running, swimming or playing soccer with friends), duration (minutes per week) and time throughout the year (months per year). These items were combined in an index reflecting minutes per week with unorganized sports activities.

Finally, participants were asked about extracurricular sports activities at school. Again, type of sports activity, duration (minutes per session) and frequency (times per week) were obtained, and minutes were multiplied with 8.5/12 to account for vacations.

A PA activities index reflecting the sum of minutes with PA in sports clubs, leisure-time sports activities, and extracurricular sports activities was calculated and used in the later analyses.

### 2.3. Potential PA Correlates

A total of 2133 variables and indices were measured during MoMo and KiGGS and were used as potential correlates of PA for this study. According to literature, we decided to use the term “correlate” to describe the statistical associations between measured variables and PA [33]. Unfortunately, describing the methodology and sources of each and every correlate that has been used in this study would go beyond the scope of this paper. References to the methodology of correlates that are reported in the result section of this paper are presented in Table 1 and Table 3. We divided the potential correlates into 34 context themes and an overview with references to detailed methods about the measured correlates can be found in secondary literature about MoMo and KiGGS [31,34,35]. The different context themes are: Context of examination (e.g., season), place of residence (e.g., population), socio-economic status (e.g., household’s net income), pulse and hearth-rate (e.g., resting pulse), motor performance measured at KiGGS (e.g., stand and reach), motor performance measured at MoMo (e.g., push-ups), anthropometry measured at KiGGS (e.g., skin fold thickness), migration background (e.g., language spoken at home), family situation (e.g., number of siblings), parents (e.g., mother’s body weight), medical health status (e.g., diagnosed hyperactivity), pain (e.g., back pain), development (e.g., month of baby’s first steps), quality of life (e.g., self-esteem (KINDL-questionnaire)), mental health (e.g., peer problems (strength and difficulties questionnaire: SDQ)), housing situation (e.g., living space), media usage (e.g., television consume per day), school (e.g., last mark in German lesson), alcohol and tobacco usage parents (e.g., mother’s smoking), family education (e.g., mother’s education), health status consequences (e.g., feeling tired), childhood (e.g., victim of violence), alcohol and tobacco usage child (e.g., number of cigarettes per day), chronic diseases (e.g., scoliosis), blood status (e.g., erythrocyte volume), acute disease and allergies (e.g., hay fever), other childhood circumstances (e.g., monthly allowance), diet (e.g., number of seafood dishes per week), insurance (e.g., type of health insurance), visits to the doctor (e.g., last visit at the orthopedist), surgeries (e.g., lifetime number of surgeries), medication (e.g., homeopathic products), urine status (e.g., fluoride) and PA of parents (e.g., father’s membership in a sports club).

The available data covers the important fields of potential PA correlates that were categorized into categories that have been postulated in previous literature: Demographic, psychological and behavioral, biological, social and physical environmental factors [20,26].

### 2.4. Ethics Approval and Consent to Participate

This study was approved by the ethics committee of the State Chamber of Physicians of Baden-Wuerttemberg (Germany). The KiGGS and the MoMo studies were approved by the Charité/Universitätsmedizin Berlin ethics committee and the Federal Office for the Protection of Data and were conducted according to the Declaration of Helsinki. All participants of the MoMo study gave their written consent to participate and were informed in detail about the study and data management by the Robert Koch Institute. Parents gave their written consent for minors and the presence of a legal guardian was mandatory under the age of 15.

## 3. Statistical Methods & Modelling

In order to identify the most meaningful correlates of PA, a three-step variable selection method was used. In the first step, the data was prepared and in step two and three least absolute shrinkage and selection operator (LASSO) regressions were conducted to rank potential correlates according to their power in predicting PA. To conduct LASSO regressions, the CATREG function of IBM SPSS 24 was used. LASSO is based on shrinkage estimation and shrinks unstable estimates to zero to exclude variables without the need for formal statistical testing [36]. LASSO also handles the multicollinearity problem [37] and has been welcomed in the literature for variable selection [38], especially when high numbers of covariates have to be considered [39].

*Step 1: Preparing the Data*

During a preliminary screening, 979 variables that were only measured in a small portion of participants were excluded. A total of 1154 remaining items and indices were then combined in 34 context related themes. Some examples of different themes are anthropometrics, motor performance, social background and status, family characteristics and climate, psychological peculiarity, blood screening, and residential neighborhood.

Metrical variables were translated into categorical variables by using practical implication whenever possible (for example body mass index (BMI)) or dividing into five percentile-based groups if no practical implication was available (for example media use, birth weight). Missing values among covariates could not be assumed to be missing completely at random (MCAR) or missing at random (MAR), as, for example, questions about the household’s income are selectively missing in families with very low and very high income. Hence an extra category for missing values was defined for each potential predictor. This technique of dealing with data not missing at random (NMAR) in covariates is suggested when the aim of the regression model is creating predictors and the reason for missing data is the person refusing to answer [40].

*Step 2: Context Theme Related Models*

For each of the 34 context themes and separately for boys and girls, a multivariate regression model with least absolute shrinkage and selection operator (LASSO) and 10-fold cross-validation was conducted to rank variables according to their power of predicting PA. The top four significant predictors of each context theme were determined. To determine a practicable number of predictors for the final model in step 3, we merged those top 136 predictors of into ten larger context themes and ran LASSO regression models again. This reduced the total number of potential predictors to 28 for boys and 29 for girls.

*Step 3: The Final Model*

After the most potential predictors were identified in step 2, we included them in a final multivariate regression model using LASSO and 10-fold cross-validation to obtain the optimal PA predictive factor subset. The CATREG function of IBM SPSS determines the optimal factor subset by choosing the model whose R² cannot be further significantly increased by adding another factor.

## 4. Results

### 4.1. Predictors of PA among Boys

The variable selection in step 2 revealed 28 main predictors for PA among boys (Table 1).

**Figure 1:**
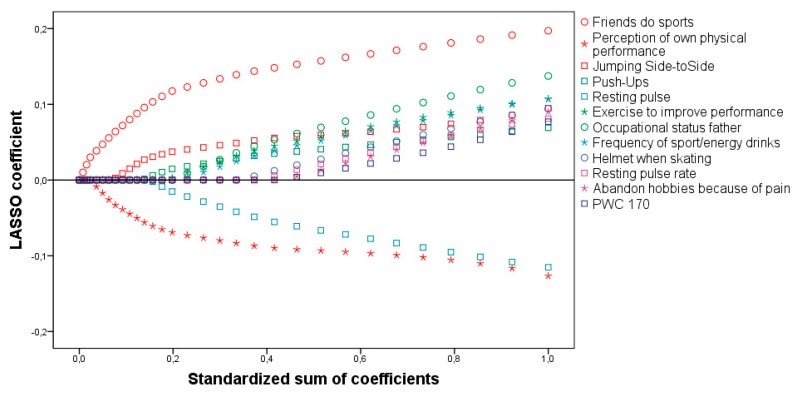
LASSO paths of the final model for boys.

The selected predictors cover the different categories of demographic, psychological, biological, social and environmental factors. For example, the age of the person, the number of completed push-ups during 40 s and the type of the family’s health insurance. From the 28 selected predictors, a total of 19 predictive factors (Table 1 marked with stars) were selected as the optimal factor subset by LASSO in step 3. Those 19 predictors explained a total of 33% of the variance of PA (R² = 0.33). LASSO paths of the final model are shown in Figure 1.

The answer to the 5-point item whether many of the participant’s friends exercise (peer modeling) turned out to be the most meaningful predictor for PA among 6 to 17-year-old boys. Followed by physical self-concept, resting heart rate and two motor performance results: The number of correct side-to-side jumps on a jumping mat during 15 s and the number of completed push-ups during 40 s. Table 2 shows the descriptive statistics of the PA index differentiated by the categories of four top predictors.

The descriptive statistics in Table 2 show that the different categories of the top-ranked predictors considerable differentiate according to the PA index. Whereas boys who report having no friends that exercise participate in sports for only 41.1 min per week, boys who report that most of their friends engage in sports participate in sports for an average of 274.7 min per week. Besides that, for every increase of one unit of the physical self-concept item, the PA index increases by about 80 min. Performance in the jumping sideways task also shows a clear relation to the PA index with a difference of about 3 h of weekly PA between the lowest and highest group of performance. Additionally, an increased resting heart rate strongly accompanies with a decreasing PA index. Whereas boys who had a resting heart rate below 64 engage for an average of 313.2 min in PA, boys who had a resting heart rate of more than 93 beats engage only for an average of 160.5 min.

### 4.2. Predictors of PA among Girls

Among girls, a total of 29 main predictors were selected in step 2 (Table 3).

**Figure 2:**
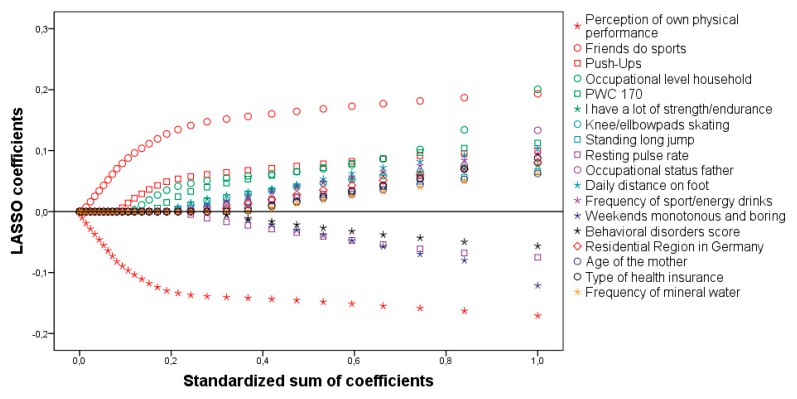
LASSO paths of the final model for girls.

Similar to the results of the boys, the selected predictors cover the different factor-categories. 18 predictive factors (Table 3 marked with stars) were selected by LASSO in step 3 and explained a total of 24.9% of PA variance (R² = 0.249). LASSO paths of the predictors in the final model ranked by relevance are shown in Figure 2.

Among 6 to 17-year-old girls, the physical self-concept according to a 5-point scale from “very good” to “not good” was the most meaningful predictor for PA. The 5-point item whether many of the participant’s friends exercise (peer modeling) is also an important predictor of girls’ PA, followed by the number of push-ups and the occupational level of the main earner in the household. Table 4 shows the descriptive statistics of the PA index differentiated by the categories of the top predictors.

Table 4 shows that every unit drop in the physical self-concept item is accompanied by a considerable reduction of weekly exercise. Girls who rate their own physical performance as “very good” exercise for about 286.0 min a week, whereas girls who rate their performance as “not good”, only engage for 59.0 min a week. Similarly to the case for boys, girls who report having many friends that exercise (peer modeling) are more likely to engage in PA and girls who report having no friends that exercise, only exercise for about 62.8 min a week. Performance in the push-up task also shows a difference of about two hours of weekly PA between the lowest and highest group of performance. Additionally, the occupational level of the household’s main earner according to a classification of professions [31] shows a considerable correlation with weekly exercise. Whereas girls from households with a low occupational level engage in PA for an average of 114.9 min, girls from moderate or high levels engage for an average of 161.8 or 175.9 min, respectively.

## 5. Discussion

Identifying correlates of PA is considered to be of public health significance because such knowledge is necessary to tailor efficient interventions that increase the proportion of people who meet health-related PA guidelines [26]. However, different reviews from the past 25 years have come to discrepant conclusions regarding correlates of PA [26,47,48]. Whereas selectivity in the type of investigated correlates may be one reason [49], correlates of PA may also be time-, region- and outcome-dependent, influenced by behavioral, social and cultural changes over time. Therefore, constantly updated empirical data over a wide range of potential correlates in large and representative samples like in the present study is needed to keep the knowledge about meaningful PA correlates up-to-date. Our study revealed that among 6 to 17-year-old boys and girls living in Germany peer modeling and physical self-concept are the most meaningful predictors of PA, followed by measured physical fitness and variables related to social status. Although they were mentioned in a variety of studies [19,20] the top predictors did not differ between boys and girls in our study.

Our results support previous research that found significant correlates in a wide range of different categories like demographic, psychological and behavioral, biological, social and physical environmental [26,48]. Even though our approach was of an explorative nature and built context themes to handle the huge amount of variables, no confirmatory decisions were involved in the variable selection, while the top three predictors for both girls and boys cover the main categories of correlates which have been identified in ecological models of health behaviors before [18].

Besides selecting the best factor subset to predict PA, the method used in our study also ranks variables according to their power of prediction. In the review by Sallis et al. [20], the most consistent correlate of PA was peer influence and peer support was the only significant correlate of objectively monitored PA. The fact that peer modeling was also selected as the most meaningful predictor in boys and the second most important predictor in girls by our explorative LASSO approach speaks quite strongly, first for the LASSO method to select meaningful correlates and second for peer influence being the most important correlate of PA nowadays. In both, boys and girls, social environmental and psychological factors were the best predictors for PA, followed by biological factors. Demographical and physical environmental factors only played a subordinate role in our study and only the region in Germany made it into the final model for boys.

In the systematic review of reviews performed by Sterdt et al. [48] that includes reviews between 2000 and 2009, 16 correlates were identified which were consistently associated with PA of children and/or adolescents aged 3 to 18 years: gender, age, ethnicity, parental education, family income, socioeconomic status, perceived competence, self-efficacy, goal orientation/motivation, perceived barriers, participation in community sports, parental support, support from significant others, access to sport/recreational facilities and time outdoors [48]. Our results match those from Sterdt et al. [48] very well but add objectively measured fitness as an important predictor of PA—in addition to perceived fitness. One reason for the fact that objectively measured fitness is not pronounced in most reviews about correlates of PA may be the fact that fitness has not been measured objectively in large epidemiological studies because it is time-consuming and costly to measure and requires a lot of effort from both investigators and subjects.

### 5.1. Demographic Factors

Concerning demographic factors, gender is known to be an important correlate for PA with age and cultural-related effect sizes [7,11,12] and we accounted for that by stratifying our sample in boys and girls. Other than that, no demographic factor was selected by LASSO for the final model, even though, age, migration background and place of birth were selected as significant predictors in step two of our variable selection. The reason for this may be that migration background and place of birth are both mediated by social and economic status such as the occupational status of the father in boys and girls and the household’s income (occupational level of the main earner) as well as the type of health insurance in girls. Most people who emigrate to Germany come from economically and educationally worse regions but there are also many highly educated specialists who migrate to Germany. Therefore, it is a reason to suspect that social factors like the occupational status are better predictors for PA than time and cultural depended demographic factors like migration background.

Interestingly, the age at measurement did not find its way into the final model. This may be explained by the fact that age as a variable has no explanatory value itself, is not causally effective, and is trivially explained by personal, social and physical environmental factors, in the present study most likely by the performance in the fitness tests.

### 5.2. Psychological and Behavioral Factors

In boys and girls, the overall perception of the own fitness and the 5-point ordinal item “Do you have a lot of strength/endurance?” from the physical self-concept scale by Stiller et al. [41] were selected as meaningful predictors of PA. These results are in line with other studies which identified self-efficacy as a consistent positive correlate and determinant of PA in children and adolescents [49,50]. In the review of Bauman et al. [49], perceived behavioral control in general perceptions of the ability to be physically active was a determinant in adolescents, but the evidence was inconclusive in children. Since we cannot assume causality from our results, we cannot conclude that interventions that only increase the physical self-concept will increase PA. However, we state that PA interventions in youths should focus on an increase in the physical self-concept, especially the self-perception of strength and endurance, at least as a secondary objective because it is so strongly correlated with habitual PA and may be crucial for maintaining sufficient levels of PA over time.

In our study, the behavioral disorders score was selected for the final model only among girls and fewer disorders were accompanied with more PA. The findings of perceived barriers to PA in children are also inconsistent [49]. Only among boys, the answer to the question “Do you abandon hobbies because of pain?”, a 5-point ordinal item from the KiGGS survey [31] made it into the final model. However, it is questionable whether this item should be assigned to psychological or biological barriers.

Similar to perceived barriers, the findings for valuing PA for their own health status are inconsistent in youth [49]. In our study, items which reflect the health status as a motif for being physically active were not selected as meaningful predictors. However, improving the physical performance as a motif to exercise made it into the final model for boys. Among girls, behavioral factors which are linked to a generally healthy lifestyle like the frequency of consuming mineral water and the daily distance traveled on foot were selected for the final model, but no motivational items. These results suggest that physical performance as a motif for PA is more pronounced in boys, whereas girls who often reject participation in competitive sports [51], do more often exercise as part of a healthy lifestyle.

### 5.3. Biological Factors

Besides genetic factors such as ethnicity, physical conditions like anthropometry, chronic disease, and physical fitness are considered as biological factors that may be correlated with PA behavior [49]. It is recently discussed that overweight and obesity show a bidirectional causal relation to PA [49,52]. However, in a wide range of large epidemiological studies, no relationship was found for BMI and other anthropometric measures with PA in children or adolescents [49]. One rationale for this finding may be the fact that healthy individuals with normal weight tend to increase their BMI when engaging in sports because of a gain in muscle mass [53]. In our study, body fat percentage derived from skinfold thickness was selected as one of the 29 most important predictors of PA in girls but was not selected for the final model that accounts for multicollinearity with other factors. The most important predictors among boys and girls were factors related to fitness such as the resting heart rate and motor performance which are very likely to cover the explanatory power of anthropometric measurements. Some health-related variables and biological barriers like the occurrence of pain during PA or the respiratory disease acute spasmodic laryngitis (croup) were selected during step two but not in the final model. An explanation could be the fact that biological barriers to PA and physical fitness show high amounts of multicollinearity when explaining PA. Physical performance tests may simply be the better predictors for PA, even though biological barriers could be the causal factors while physical fitness is only a mediator.

### 5.4. Social Environmental Factors

In the review of reviews published by Biddle et al. in 2011 [51], the authors found significant social and cultural correlates for PA in eight out of nine reviews. The studies focused mainly on parental, siblings and peer role models, behavior and support. Although there is still much uncertainty about the underlying mechanisms of social factors and especially of parental support [54,55], authors state that parental support plays an important role in young people’s PA [51,55]. Whereas social support from the parents seems to be more important in younger ages [56] and from the father [51], social support from peer groups becomes more important in later stages of adolescence [57,58,59]. In our study, peer modeling turned out to be the most important predictor of PA in boys and the second most important predictor in girls. Role modeling of the parents or siblings was not selected in our model with the exception of the younger age of the mother standing for higher PA among girls. Parental support such as driving the child to sports facilities and instrumental support was not selected but may have been covered by variables of the social-economical-status such as the occupational status of the father in boys and girls and the occupational level of the main earner among girls. According to our results, socio-economic status has been identified as an important correlate of PA in children and adolescents [33,50,51].

### 5.5. Physical Environmental Factors

Although the MoMo PAQ contains a differentiated scale to assess environmental correlates of PA [34] the region in Germany where the participants live was the only variable that was selected as an important predictor of PA in step 2. In Germany, participation in sports clubs is lower in the states of former East Germany compared to other regions [60].

Studies that focus on physical environmental factors as correlates of PA identified walkability, traffic speed, and volume (inversely), land-use-mix (proximity of homes and destinations such as shops), residential density, and access or proximity to recreation facilities as the most robust correlated for children and adolescents [49,61]. General environmental walkability may be pronounced by the overall distance traveled by foot, which was selected by LASSO in our study for the final model among girls. However, our results suggest that the physical environmental factors that have been measured in our study are less important correlates of PA when compared to biological and social environmental factors.

### 5.6. Strengths and Limitations

The aim of our study was to identify the most meaningful predictors of PA within the framework of the MoMo-study by selecting them according to their power of explaining PA. Since no confirmatory decisions have been made, mediator variables may have been selected over causal variables because they mediate a sum of causal and/or unmeasured variables and explain more variance than single causal factors. Therefore we cannot conclude causality from our results. However, the descriptive results of our study show that the identified correlates of PA strongly differentiate between more and less active children and adolescents.

PA was assessed by retrospective self-reports. This method has various limitations, including recall bias and social desirability [13]. Measuring PA by objective methods such as accelerometers is more accurate in most types of PA, but is always limited to a short time interval and children drop it for some sports like swimming, martial arts and sometimes even curricular sport at school. Additionally, accelerometers are responsive and trigger socially desired behavior [62]. Interestingly, studies that apply objective methods of PA assessment in children and adolescents have generally identified fewer associations with correlates and accounted for less variance than those that have relied upon self- or proxy-reports [25].

Lastly, MoMo and KiGGS put a huge effort in collecting representative data from a total of 167 sample points across Germany and paired with a total number of 1154 gathered potential correlates of PA the evidence of our results is suggested to be high. For the first time, this study provides knowledge about correlates of PA in a representative German sample and considers a number of potential correlates that have, to the best of our knowledge, only be considered in reviews about multiple, time and methodologically heterogeneous conducted studies before.

## 6. Conclusions

Out of 1154 different demographic, psychological, behavioral, biological, social and physical environmental factors, this study identified peer modeling and the perception of the own physical fitness as the strongest correlates of PA in boys and girls aged 6 to 17 living in Germany. Our results support popular socio-ecological models of behavior that posit influences from different, personal, social and physical environmental factors [26]. Therefore, we can confirm that interventions must target changes in variables from all levels of influence to achieve substantial behavior change which has also been postulated in previous research [25,51]. Additionally, we add a prioritization on the social environment and self-perception of the own physical fitness for intervention programs aiming to increase physical activity in children and adolescents in Germany. The fact that physical environmental factors played a subordinate role in our results is clearly time-dependent and not generalizable beyond Germany. Therefore, we strongly recommend repeatedly tracking correlates of PA, at least every 10 years, from representative samples in order to tailor ideal, contemporary PA interventions.

## Figures and Tables

**Table 1:** Selected main predictors for PA among boys.

Category	Variable	Details
Demographic	Age	Age at measurement
	Migration background	2-point item from the KiGGS survey: “yes”, “no” [31]
	Place of birth is Germany	2-point item from the KiGGS survey: “yes”, “no” [31]
Psychological and behavioral	* “Do you have a lot of strength/endurance?”	5-point ordinal item from the physical self-concept scale by Stiller et al. [41]
	Hours of sleep during the last night	Metrical item from the KiGGS survey [31]
	“During the past week, I had fun and laughed a lot”	5-point ordinal item from the KINDL scale to assess health-related quality of life [42]
	* Perception of the own physical performance	5-point ordinal item from the physical self-concept scale by Stiller et al. [41]
	* “Do you abandon hobbies because of pain?”	5-point ordinal item from the KiGGS survey [31]
	* “I do exercise to improve my physical performance”	5-point ordinal item from the MoMo survey [35]
	* Frequency of sport or energy drinks	9-point ordinal item from “never” to “more than 5-times a day” [31]
	* “Do you wear a helm during inline skating”	3-point item from the KiGGS survey: “yes”, “no”, “does not skate” [31]
Biological	* Resting heart rate	Measured during KiGGS [31]
	* Push-ups	Number of Push-ups in 40 s [34]
	* PWC 170	Physical work capacity at pulse 170 [34]
	* Jumping sideways	Number of correct side-to-side jumps during 15 s [34]
	Mean arterial pressure	Measured during KiGGS [31]
	Waist to hip ratio	Measured during KiGGS [31]
	Thyroid volume	Measured during KiGGS [31]
	Mean corpuscular volume	Result of the blood analysis [31]
	Acute spasmodic laryngitis	Respiratory disease identified during KiGGS [31]
	Infantile development during preschool age	3-point item from the KiGGS survey: “fast”, “normal”, “slow development” [31]
	Pain during physical activity	2-point Item from the KiGGS survey: “yes”, “no” [31]
Social environmental	* “Do many of your friends engage in sports?”	4-point ordinal item from a social support and model behavior scale [43]
	Type of health insurance	8-point nominal item covering the type of health insurances in Germany [31]
	* Occupational status of the father	6-point nominal item from the KiGGS survey: “full-time”, “part-time”, “not working, e.g., retired”, “unemployed”, “vocational training”, “temporary exemption” [31]
	Main residence	8-point nominal item from the KiGGS survey: “parents”, “father”, “mother”, “father & partner”, “mother & partner”, “grandparents”, “foster parents”, “institution”. [31]
	Type of school	9-point nominal item covering the type of schools in Germany [31]
	* “Do you wear a helm during inline skating”	3-point item from the KiGGS survey: “yes”, “no”, “does not skate” [31]
Physical environmental	Residential Region in Germany (7 regions)	7-point nominal item (Nielsen-Areas) [31]

*: variable selected for the final model by LASSO regression.

**Table 2:** Descriptive statistics of top predictors of PA among boys aged 6 to 17.

Predictor	*n*	PA index Mean ± Standard Deviation [min/week]
“Do many of your friends exercise?”		
most of them	845	274.7 ± 216.0
some	533	182.5 ± 181.0
only a few	209	131.6 ± 192.7
none	23	41.1 ± 47.7
missing value	191	214.3 ± 186.0
Perception of physical performance		
very good	256	336.6 ± 265.5
good	454	255.7 ± 220.9
average	195	165.1 ± 164.8
not very good	25	84.1 ± 111.7
not good	2	130.0 ± 183.8
missing value	6	123.1 ± 133.2
missing (age < 11)	863	186.9 ± 170.1
Jumping sideways [jumps]		
≥39	185	330.5 ± 250.7
35–39	270	269.6 ± 235.4
27–34	448	239.6 ± 212.6
19–26	452	189.2 ± 167.8
14–18	259	178.1 ± 176.4
<14	173	141.7 ± 153.9
missing value	14	106.1 ± 94.9
Resting heart rate [beats/min]		
<64	166	313.2 ± 239.9
64–69	255	258.9 ± 218.4
70–78	454	231.0 ± 205.2
79–85	407	208.7 ± 195.7
86–92	313	181.7 ± 170.4
≥93	197	160.5 ± 204.2
missing value	9	279.5 ± 168.5

**Table 3:** Selected main predictors for PA among girls.

Category	Variable	Details
Demographic	Age	Age at measurement
	Migration background	2-point item from the KiGGS survey: “yes”, “no” [31]
Psychological and behavioral	* “Do you have a lot of strength/endurance?”	5-point ordinal item from the physical self-concept scale by Stiller et al. [41]
	* Perception of the own physical performance	5-point ordinal item from the physical self-concept scale by Stiller et al. [41]
	* Frequency of sports or energy drinks	9-point ordinal item from “never” to “more than 5-times a day” [31]
	* Amount of mineral water consumed	9-point ordinal item from “never” to “more than 5-times a day” [31]
	* Daily distance traveled on foot	6-point item from “I hardly ever travel by foot” to “10 km or more” [35]
	Subjective health status	5-point item from “very good” to “very bad” according to the World Health Organization (WHO) [44]
	* Behavioral disorders score	Total behavioral disorders score of the strength and difficulty questionnaire (SDQ) [45]
	Amount of margarine consumed	9-point ordinal item from “never” to “more than 5-times a day” [31]
	* “My weekends are often monotonous and boring”	4-point item from the family climate scale by Schneewind [46]
	* “Do you wear a knee or elbow pads during inline skating”	3-point item from the KiGGS survey: “yes”, “no”, “does not skate” [31]
Biological	* Resting pulse rate	Measured during KiGGS [31]
	* Push-ups	Number of Push-ups in 40 s [34]
	* PWC 170	Physical work capacity at pulse 170 [34]
	Mean corpuscular volume	Metrical result of the blood analysis [31]
	Jumping sideways	Number of correct side-to-side jumps during 15 s [34]
	* Standing long jump	Performance in the standing long jump task in centimeters [34]
	Mean folic acid in erythrocytes	Metrical result of the blood analysis in nanograms per milliliter [31]
	Body fat percentage	Metrical result of the measurement of skinfold thickness [31]
	Pain during physical activity	2-point item from the KiGGS survey: “yes”, “no” [31]
	Number of visits of the pediatrician	Metrical item (visits during one year) [31]
	Number of visits of the dermatologist	Metrical item (visits during one year) [31]
Social environmental	Type of school	9-point nominal item covering the type of schools in Germany [31]
	* “Do many of your friends engage in sports?”	4-point ordinal item from the social support and model behavior scale by (Reimers et al., 2012)
	* Occupational status of the father	6-point nominal item from the KiGGS survey: “full-time”, “part-time”, “not working, e.g., retired”, “unemployed”, “vocational training”, “temporary exemption” [31]
	* Type of health insurance	8-point nominal item covering the type of health insurances in Germany [31]
	* Age of the mother	Age of the mother at measurement [31]
	* Occupational level of the main earner	3-point item: “low”, “moderate”, “high” [31]
Physical environmental	* Residential Region in Germany (5 regions)	5-point nominal item from the KiGGS survey “Northwest”, Northrhine-Westphalia”, “Middle”, “East”, “Bavaria and Baden-Württemberg” [31]

*: variable selected for the final model by LASSO regression.

**Table 4:** Descriptive statistics of top predictors of PA among girls aged 6 to 17.

Predictor	*n*	PA Index Mean ± Standard Deviation [min/week]
Perception of physical performance		
very good	136	286.0 ± 231.0
good	385	188.9 ± 171.3
average	282	122.4 ± 157.6
not very good	73	72.4 ± 101.4
not good	5	59.0 ± 77.0
missing value	849	130.5 ± 136
missing (age < 11)	8	111.2 ± 161.9
“Do many of your friends exercise?”		
most of them	581	201.2 ± 177.2
some	580	138.0 ± 155.0
only a few	340	102.4 ± 143.0
none	60	62.8 ± 95.0
missing value	177	157.5 ± 146.7
Number of push-ups		
<7	144	103.5 ± 125.6
7–8	212	126.6 ± 152.0
9–10	345	105.4 ± 120.9
11–13	542	165.0 ± 163.6
14–15	255	170.7 ± 160.0
≥16	235	220.7 ± 212.1
missing value	5	101.0 ± 73.9
Household’s occupational level		
low	515	114.9 ± 141.9
moderate	527	161.8 ± 173.5
high	648	175.9 ± 166.7
missing value	48	104.9 ± 133.1

## Declarations

The datasets used and/or analyzed during the current study are available from the corresponding author on reasonable request.

## Abbreviations

PA	Physical Activity
MoMo	Motorik-Modul Study
MoMo-PAQ	Motorik-Modul Study physical activity questionnaire
KiGGS	German Health Interview and Examination Survey for Children and Adolescents
RKI	Robert Koch Institute
GESIS	German Centre for Surveys, Methods, and Analysis
BMI	Body Mass Index
LASSO	Least absolute shrinkage and selection operator
